# Supplementary material for: Predicting visual function by interpreting a neuronal wiring diagram
Source: Nature. 2024 Oct 2;634(8032):113–23. doi: 10.1038/s41586-024-07953-5 (PMC11446822; doi:10.1038/s41586-024-07953-5)
Supplement: Supplementary file 5 — CRF and ERF predictions for individual Dm3 cells. The CRF is predicted for each target cell by mapping the top five monosynaptic pathways from hexel source types to the target Dm3 type. The ERF is predicted using disynaptic pathways that pass through the top ten intermediary types (ranking from Extended Data Fig. 7). As a set of disynaptic pathways passes from multiple hexel source types through each intermediary to the target, for brevity only one representative starting from a single hexel type is shown, the pathway in the set with maximal strength (red line, Extended Data Fig. 7). The centre of the Tm1–Dm3 CRF (Methods) is indicated by the cyan star, to aid comparison of locations across maps. Next to each map are the maximum and sum over all hexels, in units of 0.01%. In the last panel, all ellipse approximations are shown together for comparison, scaled up by ×3 relative to maps. The dashed ellipse approximates the Tm1–Dm3 CRF, and scale bars indicate one lattice constant and p and q axes. Each file name contains the ID of the target cell in v783. [file 41586_2024_7953_MOESM5_ESM.zip › DataS3/Dm3p/720575940632890937.pdf]

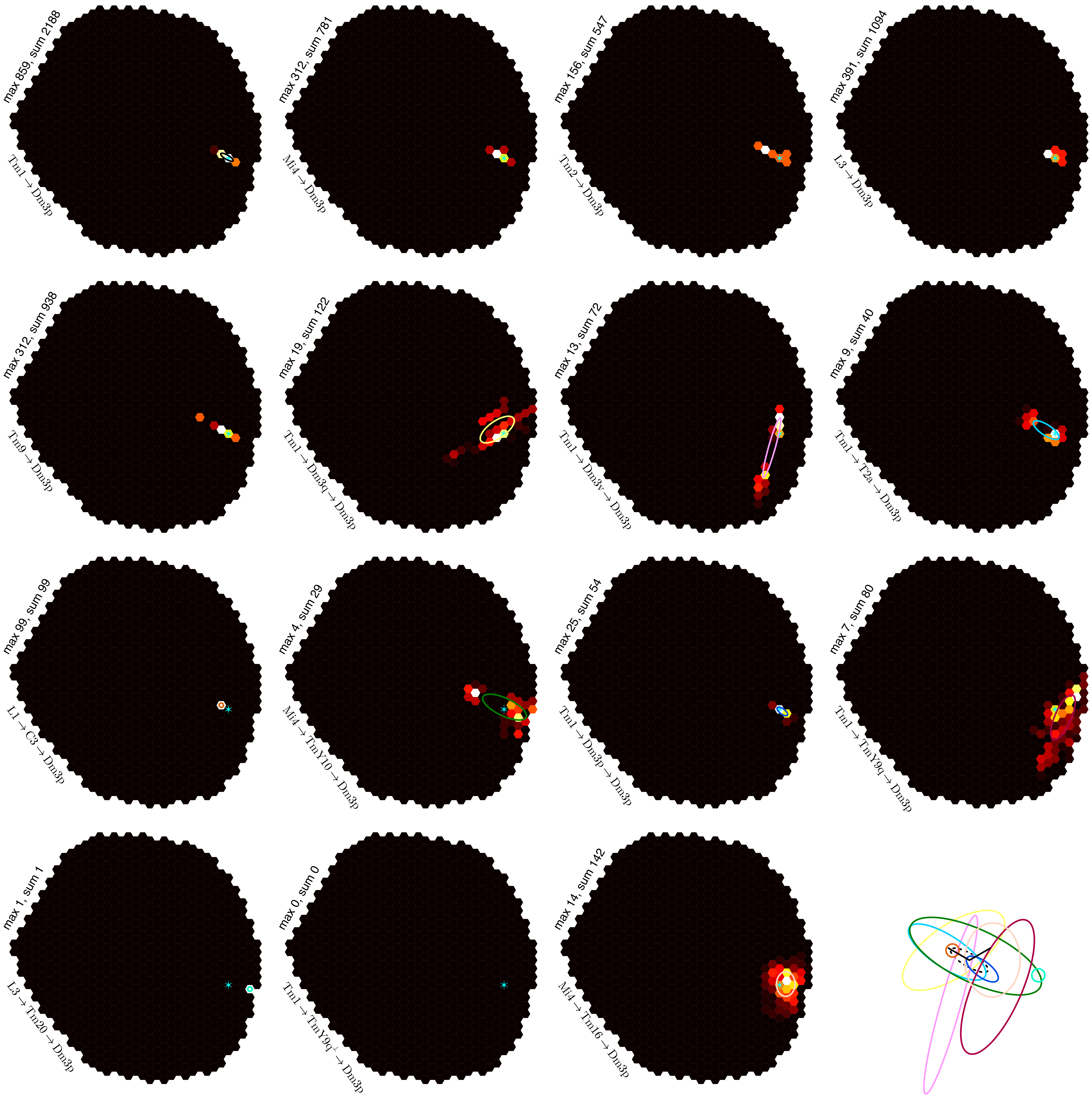
max 859, sum 2188
Tm1 → Dm3p
max 312, sum 781
Mi4 → Dm3p
max 156, sum 547
Tm2 → Dm3p
max 391, sum 1094
L3 → Dm3p
max 312, sum 938
Tm9 → Dm3p
max 19, sum 122
Tm1 → Dm3q → Dm3p
max 13, sum 72
Tm1 → Dm3v → Dm3p
max 9, sum 40
Tm1 → T2a → Dm3p
max 99, sum 99
L1 → C3 → Dm3p
max 4, sum 29
Mi4 → TmY10 → Dm3p
max 25, sum 54
Tm1 → Dm3p → Dm3p
max 7, sum 80
Tm1 → TmY9q → Dm3p
max 1, sum 1
L3 → Tm20 → Dm3p
max 0, sum 0
Tm1 → TmY9q⊥ → Dm3p
max 14, sum 142
Mi4 → Tm16 → Dm3p
